# Precision and Reliability of Tightly Coupled PPP GNSS and Landmark Monocular Vision Positioning

**DOI:** 10.3390/s20051537

**Published:** 2020-03-10

**Authors:** Menglin Pang, Christian Tiberius

**Affiliations:** Department of Geoscience and Remote Sensing, Faculty of Civil Engineering and Geosciences, Delft University of Technology, Stevinweg 1, 2628 CN DELFT, The Netherlands; menglin.pang@samsung.com

**Keywords:** GNSS, Precise Point Positioning (PPP), monocular camera, landmark, sensor integration, precision, reliability

## Abstract

This paper presents an approach to analyse the quality, in terms of precision and reliability, of a system which integrates—at the observation-level—landmark positions and GNSS measurements, obtained with a single camera and a digital map, and a single frequency GNSS receiver respectively. We illustrate the analysis by means of design computations, and we present the actual performance by means of a small experiment in practice. It is shown that the integration model is able to produce a position solution even when both sensors individually fail to do so. With realistic assumptions on measurement noise, the proposed integrated, low-cost system can deliver a horizontal position with a precision of better than half a meter. The external reliability of the integrated system is at the few decimetre-level, showing that the impact of undetected faults in the measurements, for instance incorrectly identified landmarks in the image, on the horizontal position is limited and acceptable, thereby confirming the fault-robustness of the system.

## 1. Introduction

The goal of this study is to develop a tightly coupled integration model of Global Navigation Satellite System (GNSS) and monocular vision (single camera) measurements, and to propose a method to analyse the positioning performance in particular in terms of precision and reliability, for instance for the application of advanced car navigation and further levels of automation of road-vehicles, such as assisted driving—this paper is largely based on the research in [1].

We will use single frequency Precise Point Positioning (PPP) GNSS, and a single camera together with a High Definition (HD) map [2] to observe well identifiable objects, which we will call landmarks. These two sensors are complementary, as the camera relies on the availability of landmarks around the vehicle, which is typically large in urban areas, and low in rural areas, and GNSS performs generally well in the latter.

Precise Point Positioning (PPP) is a positioning technique which utilizes un-differenced pseudorange and carrier phase measurements with the aid of GNSS data products from a global network of reference stations providing precise satellite orbits and clocks [3,4,5]. We use single-frequency PPP [6]. This technique has been applied for lane level positioning [7,8].

A vision sensor can be used to evaluate vehicle motion [9]. It can track the features in a sequence of images, taken over a time interval, and uses previous absolute position information to estimate position and attitude [10]. To correct for the drift in visual odometry [11] found that a proper density of landmarks is critical to compensate for the accumulated errors in visual odometry. Reference [12] proposed fusion of inertial sensors with monocular vision, using camera motion. With the aid of a digital map, previously surveyed positions of landmarks, which the vehicle captures by its vision system, can be retrieved from the map database, and be used in vehicle state estimation. Reference [13] proposed the integration of GNSS and laser-scanner measurements, using for the latter specifically a range and angle measurement per landmark (so-called polar measurements), in order to deal with GNSS signal blocking in forests and urban areas. Landmarks facilitate the feature matching process with high precision due to their simple geometric form, lower storage volume, and lower matching errors of their shape and texture [11]. Reference [14] analyse feature matching errors, when tracking objects over time, in a series of images. Reference [15] showed the feasibility of integrating GNSS range measurements with stereo image measurements of landmarks, and [16] the integration of low-cost GNSS and a monocular camera. Reference [17] presented a model integrating inertial and vision measurements, and Reference [18] does so for GNSS and vision-aided MEMS inertial measurements. Reference [19] shows field-test results of a tightly coupled RTK-GNSS, MEMS INS and monocular vision system. Reference [20] used a selective integration of raw measurements from different sensors, aiming to recognize poor environments. Reference [21] developed a real-time positioning method by fusing GPS and vision-based lane mark detection in urban areas. Reference [22] showed that adding vision to GNSS can improve Receiver Autonomous Integrity Monitoring (RAIM) during the landing phase of an aircraft.

Though many papers elaborate on the integration of GNSS and vision measurements, and show experimental results, none were found to present—in relation to the design of the system—a detailed analysis of the quality of the integrated system in terms of precision and reliability. Precision describes the usual, or nominal spread in the resulting position solution due to ordinary measurement noise, and reliability—linked to integrity of the system—describes robustness, realized by means of statistical measurement testing, against incidental, excessive faults and anomalies.

In Section 2 of this paper, the mathematical model for both the GNSS and vision measurements is presented, as well as their integration at the measurement level. Then, the measures of interest are introduced to describe and quantify the performance of the system, in terms of precision and reliability. In Section 4 we illustrate the use of these measures and present extensive results from the design study, covering different configurations and scenarios, in order to learn and understand the strengths and weaknesses of the sensors and their integration. Finally, in Section 5 we present the results from an actual field experiment.

As this study is about the analysis of the quality of an integrated GNSS-vision-system in terms of precision and reliability, rather than developing a user-ready operational system, various simplifications were made. A local flat, horizontal road is assumed, such that the camera horizon is parallel to the local horizon—the camera is only subject to heading changes, and not to roll and pitch. The analysis is limited to single snapshot data-collection, meaning that we use the GNSS and vision measurements of just a single epoch in time. The focus is on instantaneous positioning (therefore we do not consider GNSS carrier phase measurements, which over time, may effectively ‘smooth’ the pseudorange measurements). In practice, the performance can be improved by using a time series of measurements, for instance by means of a Kalman filter. In the experiment eventually, the vision measurements in the image were not performed by an object recognition algorithm, but rather by a human operator, as well as the landmark identification.

## 2. GNSS, Vision and Integration Model

In this section we outline the mathematical model, first for GNSS single frequency Precise Point Positioning (PPP), then for the camera image measurements, and eventually for the integrated system.

### 2.1. SF-PPP Model

The mathematical model for SF-PPP measurements consists of a functional component and a stochastic component. In PPP the pseudorange observations are corrected using external data products [23,24] and chapter 25 in [25]. The implementation is based on [26]’s model. The position coordinates are in an Earth-Centered, Earth-Fixed (ECEF) frame, WGS84, or with PPP in our case, in ITRF2008 (International Terrestrial Reference Frame). In this study we use, for simplicity, pseudoranges only, though carrier phase measurements can quite easily be added in, see [26]. The linearized positioning model with *k* visible satellites is:

(1) $$\begin{array}{c} \left[\begin{array}{c} \Delta p^{k} \end{array}\right]=\left[\begin{array}{ccc} -h_{G}^{k} & u^{k} & \delta \end{array}\right]\left[\begin{array}{c} \Delta r_{r}\\ \Delta t_{r}\\ b \end{array}\right]+\epsilon_{G} \end{array}$$

the increments of receiver position and receiver clock bias $\Delta r_{r},\Delta t_{r}$ are the differences between the unknown parameter values and an initial guess, $\epsilon_{G}$ contains measurement noise, *b* is the intersystem bias (clock effect between GPS and GLONASS for instance), $h_{G}$ contains the unit direction vectors pointing from the receiver to the satellites, as row vectors, *u* is a column of ones (of dimension *k*), elements of vector δ are 1 for one constellation or 0 for the reference one.

Since the satellite orbit and clock error, tropospheric and ionospheric error are evaluated based on external products and models, their uncertainty should be included in the stochastic model. Combining their effects and the observation uncertainty, the observables’ variance matrix in the stochastic model becomes:

(2) $$\begin{array}{c} Q_{p}+J_{p}Q_{r^{s},t^{s},n,\iota }J_{p}^{T}=Q_{pp} \end{array}$$

where $Q_{p}$ contains the measurement noise, $Q_{r^{s},t^{s},n,\iota }$ includes the uncertainty of the parameters obtained from external services (and models), $J_{p}$ is the Jacobian of observation equation with respect to these parameters. The measurements are collected, in our case, by a low-cost GNSS receiver, and the value of the receiver measurement noise is set to: $\sigma_{p}$ = 0.3 m (zenith). The standard deviation is elevation angle dependent.

### 2.2. Monocular Vision Model

The mathematical model for vision measurements consists of a functional and stochastic component.

**Figure 1:**
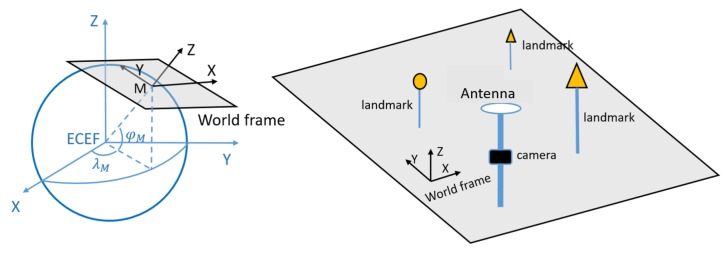
On the left: conversion from [X,Y,Z] (ECEF, in blue) to [X,Y,Z] (world frame, in black). On the right: visualization of GNSS/monocular vision set up in the world frame. The camera is not necessarily in the origin of the world frame. The world frame is converted with respect to point M with geographic coordinates $\left[\varphi_{M},\lambda_{M},h_{M}\right]$, *M* is the centre of this local topocentric reference coordinate system.

The system set-up is shown in Figure 1. A number of landmarks with pre-surveyed positions within the angle of view are captured by a single camera. Therefore, the camera pose can be derived from inputting these position measurements in the world frame in combination with the central projection model [27]. To simplify the problem, we consider only heading changes in the world frame (camera horizon aligned with local horizon, basically a flat road assumption, rotation only about the black Z-axis).

The goal of the functional model is to be able to obtain camera position and attitude (heading angle κ), by measurements of positions of landmarks in the image, which are denoted by (u,v) and expressed in pixels, and, available position (world-frame) coordinates of these landmarks (from the map). We abbreviate the camera position and attitude parameters ${\left[\kappa ,X_{0},Y_{0},Z_{0}\right]}^{T}$ as *P*, landmark position coordinates ${\left[X,Y,Z\right]}^{T}$ as *G*, and *m* denotes the number of features or landmarks. Vectors $u^{m}$ and $v^{m}$ are respectively the horizontal and vertical ’pixel’ measurements in the image, for all *m* landmarks together. The non-linear functional model of the vision measurements with respect to the camera position and attitude is symbolically expressed as:

(3) $$\begin{array}{c} y=\left[\begin{array}{c} u^{m}\\ v^{m} \end{array}\right]=H\left(P,G\right)+\epsilon_{V}=\left[\begin{array}{c} H_{u}^{m}\left(P,G\right)\\ H_{v}^{m}\left(P,G\right) \end{array}\right]+\epsilon_{V} \end{array}$$

where *y* contains the image measurements, and $\epsilon_{V}$ represents random errors. In our case we are only interested in estimating *P*, not in *G*, and we do account—in estimating *P*—for the uncertainty in the given landmark positions (in world-frame). The advantage is a lower computational load, due to reduction of the functional model dimensionality.

The image measurement noise is represented by standard deviations $\sigma_{u}$ and $\sigma_{v}$; the feature or landmark position measurements in the image are assumed to be independent, and the u-coordinate and v-coordinate of one feature are also assumed to be independent.

The landmark position uncertainty is represented by variance matrix $Q_{xx}$. In this study the available coordinates in the three directions, obtained from the map, are assumed to be independent. To take the landmark position uncertainty into consideration, one can project the uncertainty into the image measurement uncertainty $Q_{yy}$. With the variance propagation law, the projected variance for the linearized functional model is $J_{G}Q_{xx}J_{G}^{T}$, and the observation uncertainty eventually is:

(4) $$\begin{array}{c} \Delta Q_{yy}=Q_{yy}+J_{G}Q_{xx}J_{G}^{T} \end{array}$$

where $J_{G}={\left[\frac{\partial H_{u}^{m}\left(P,G\right)}{\partial G},\frac{\partial H_{v}^{m}\left(P,G\right)}{\partial G}\right]}^{T}$ is the Jacobian matrix of Equation (3) with respect to the landmark position $\left[X^{m},Y^{m},Z^{m}\right]$.

### 2.3. Integration Model

The system setup is shown in Figure 1, and we assume that the GNSS receiver and camera are mounted together with just a translation offset in the world frame, which is expressed as vector $t_{0}$. We assume that this offset accounts for a height difference between these two sensors. $P_{r}$ represents the GNSS receiver position coordinates in the world frame, and ${\left[X_{0},Y_{0},Z_{0}\right]}^{T}$ in $P_{v}$ are the camera position coordinates in the world frame respectively, $P=[\kappa ,P_{v}]$. In the integration model we introduce the height offset $t_{0}$ as an additional unknown parameter (and later, in the so-called extended integration model, we assume that it is either absent, or known, e.g., through calibration).

(5) $$\begin{array}{c} P_{r}=t_{0}+P_{v} \end{array}$$

The vehicle position coordinates are in the local topocentric coordinate system, and conversions from the local world frame to the ECEF frame ${}^{E}C_{N}$ are included in the functional model for the GNSS part, in order to produce vehicle position estimates from the observations of both sensors. The following linearized integration model is based on SF-PPP GNSS and vision:

(6) $$\begin{array}{c} \left[\begin{array}{c} \Delta p^{k}\\ \Delta u^{m}\\ \Delta v^{m} \end{array}\right]=\left[\begin{array}{ccccc} -h_{G,r_{r}}^{k}{}^{E}C_{N} & \; & \; & u^{k} & \delta^{k}\\ h_{V,P_{v}}^{m} & -h_{V,P_{v}}^{m} & h_{V,\kappa }^{m} & \; & \;\\ h_{V,P_{v}}^{m} & -h_{V,P_{v}}^{m} & h_{V,\kappa }^{m} & \; & \; \end{array}\right]\left[\begin{array}{c} \Delta P_{r}\\ t_{0}\\ \Delta \kappa \\ \Delta t_{r}\\ b \end{array}\right]+\epsilon \end{array}$$

where $\Delta P_{r},\Delta t_{r},b$ denote the GNSS receiver position in world frame, the receiver clock bias, the intersystem bias, $h_{G}\;\mathrm{and}\;\;h_{V}$ contain the vectors of partial derivatives with respect to the unknown parameters for GNSS and vision respectively, and they are the set of unit vectors from the receiver to the satellites, as row-vectors, and the Jacobian of feature or landmark measurements in the image with respect to the parameters specified in the subscript, $\left[h_{V,\kappa }^{m},h_{V,P_{v}}^{m}\right]=\frac{\partial H^{m}\left(P,G\right)}{\partial P}$.

The vision model has one orientation parameter κ to be estimated, *u* is a column of ones (with the dimension indicated by the superscript), δ contains 1’s for one GNSS constellation and zeros for the reference one (e.g., respectively Glonass and GPS). ϵ represents the measurement noise of GNSS and vision. By the conversion matrix ${}^{E}C_{N}$ from the world frame to the ECEF (WGS-84) frame, one can link the GNSS receiver position and the camera position together, and combine them into a common unknown vector $\Delta P_{r}$ in the integration model.

(7) $$\begin{array}{c} \Delta r_{r}={}^{E}C_{N}\Delta P_{r} \end{array}$$

$r_{r}$ is the receiver position in the ECEF frame used with the GNSS observations. It is emphasized that this relation (7) only holds for increments in the position vectors—it does not hold in absolute sense, due to the difference in origin. The integration model we just presented has difficulty in estimating both $Z_{0},t_{r}$ and $t_{0}$ together, as we will see in the next sections. The extended integration model assumes $t_{0}$ as given. This expands application to cases where GNSS and vision both fail individually.

The observations for the integrated model are: pseudoranges, and two-dimensional image measurements. Assuming that GNSS and image measurements are uncorrelated, we combine (2) and (4) to result in the observables’ variance matrix for the integration model.

In this section, only a concise overview has been given of the mathematical models for GNSS, vision, and the integrated system. For full details and derivations, the reader is referred to chapter 3 of [1].

## 3. Description of Performance Measures

To evaluate the performance of the above model, several quantities of interest are defined, which give further insight in the model and the interpretation of results. In this section, the concepts of these quantities are introduced, as well as their computation and interpretation.

The goal of our study is to evaluate the quality of vehicle state estimation, in terms of precision and reliability. Precision shows the influence of measurement noise propagated into the result, and the correlation coefficient measures the dependence of any two state estimators, and, reliability shows the capability of detecting faults and anomalies in the measurements by statistical testing, and their impact on the state in case they would remain undetected.

### 3.1. Precision: Standard Deviation and Correlation Coefficient

The state estimator’s variance matrix in the estimation process, $Q_{\hat{P}\hat{P}}$, is symmetric, and contains the variances on the diagonal, and covariances between the estimators elsewhere. Suppose there are two estimators ${\hat{P}}_{1}$ and ${\hat{P}}_{2}$. To obtain measures of precision and correlation, $Q_{\hat{P}\hat{P}}$, should be interpreted as: taking the square root of the diagonal elements which are the standard deviations $\sigma_{\hat{P_{1}}}$ and $\sigma_{\hat{P_{2}}}$ of the estimators (with the same units as the state parameters, e.g., a position coordinate in meters); omit the lower triangular part because of symmetry, and divide the upper part non-diagonal elements, which is the covariance $C({\hat{P}}_{1},{\hat{P}}_{1})$, by the corresponding standard deviations. The correlation coefficient of two estimators is then:

(8) $$\begin{array}{c} \rho \left(\hat{P_{1}},\hat{P_{2}}\right)=\frac{C(\hat{P_{1}},\hat{P_{2}})}{\sigma_{\hat{P_{1}}}\sigma_{\hat{P_{2}}}} \end{array}$$

(9) $$\begin{array}{c} -1\leq \rho (\hat{P_{1}},\hat{P_{2}})\leq 1 \end{array}$$

The correlation coefficient is ‘normalized’ into the interval [−1,+1], and thereby easy to interpret in a uniform way. Assuming a statistical distribution for the estimator error allows one to turn the standard deviation into an interval for the parameter, and link it to a certain probability.

### 3.2. Internal Reliability: Minimal Detectable Bias

Precision describes the quality of the position solution by means of a to-be-expected spread in the result, based on nominal conditions, with ordinary measurement noise. In a measurement system, extra-ordinary faults and anomalies may happen incidentally. A GNSS pseudorange measurement may be outlying, contain a bias, due to local environmental conditions, think of multipath, or a given in-appropriate satellite clock (correction). In this study we consider outliers in GNSS pseudoranges; each pseudorange is tested for an outlier. A vision measurement may be incorrect, possibly in poor vision conditions, with bright sun-light reflections, fog, bad weather or at night. We will consider a fault in one direction (so, the *u* or *v* image coordinate of a certain landmark is incorrect), as well as joint faults in both directions (so, both *u* and *v* image coordinates of a certain landmark are incorrect, and likely by a different amount). A cause for the latter fault could be incorrect feature matching. Reference [14] described such feature extraction and matching errors (outliers). They can easily occur with man-made objects of repetitive patterns and naturally occurring objects with self-similarity. So, in this study, we consider both one dimensional fault and two dimensional faults in image measurements.

In practice, statistical hypothesis testing is carried out in conjunction with parameter estimation, in order to be able to detect the above described faults and outliers. The nominal mathematical model, as described before, is taken as the null hypothesis, and an extended model, which includes an additional unknown bias, fault, or otherwise large error, is set to be the alternative hypothesis. By means of reliability measures we can describe the performance of this testing [28]. The Minimal Detectable Bias (MDB) describes the internal reliability, determined by matching a certain probability of false alarm α and of detection, power γ=1−β, where β is the probability is missed detection [29,30].

**Figure 2:**
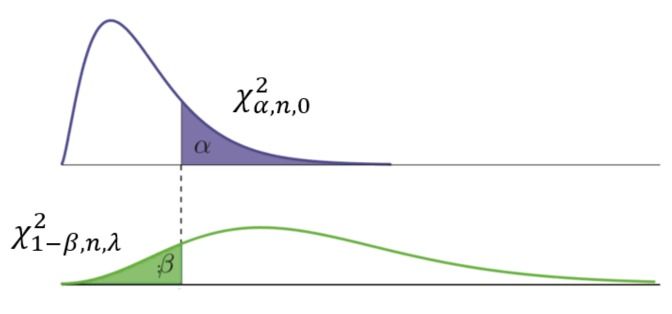
Central (top) and non-central (bottom) Chi-square distribution of test-statistic under null and alternative hypothesis respectively: false alarm (type I error) probability α and missed detection (type II error) probability β are indicated.

In order to compute the MDB, one needs to find the corresponding value of the non-centrality parameter γ, of the test-statistic distributions (under the null and alternative hypothesis), that meets the false alarm probability α and missed detection probability β (the purple and green area in Figure 2 respectively) for testing the two hypotheses. In this experiment we set the false alarm (type I error) probability α to 0.005, and identically the missed detection (type II error) probability β, as to anticipate on stringent reliability requirements for assisted driving. Once the non-centrality parameter has been found, the corresponding (one dimensional) fault or outlier magnitude can be retrieved using the observation model (the magnitude can be determined, not its sign, due to the quadratic equation involved).

**Figure 3:**
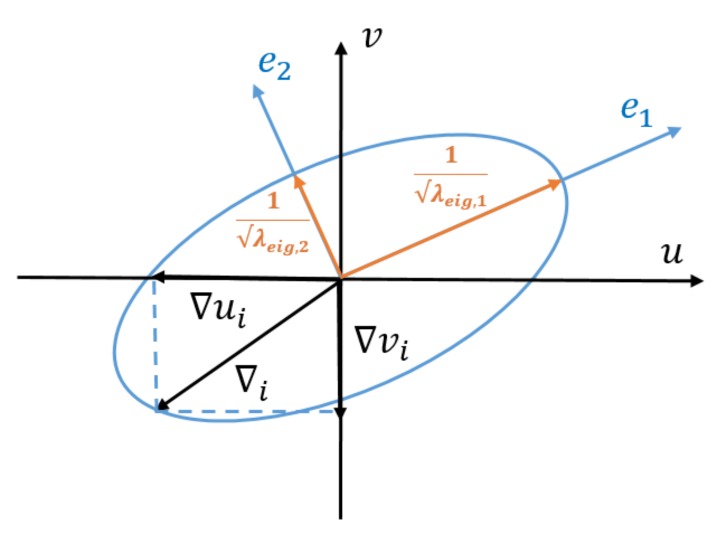
Internal reliability ellipse for a two-dimensional fault. Shown are the major and minor axes of the ellipse (in orange), and their lengths are $\frac{\sqrt{\lambda }}{\sqrt{\lambda_{eig,1}}}$ and $\frac{\sqrt{\lambda }}{\sqrt{\lambda_{eig,2}}}$. The fault magnitude, that can be detected by probability γ, ranges between these two values.

For a two-dimensional fault, the quadratic equation for the non-centrality parameter yields the border of an ellipse as a solution, see Figure 3. With the two unknown fault parameters collected in vector ▽, the ellipse follows from solving the quadratic equation ${▽}^{T}B▽=\lambda$ (for a fixed value of λ). Points $▽={\left(▽u,▽v\right)}^{T}$ on the border of the ellipse represent combinations of a large error ▽u in image measurement *u* and another large error ▽v in image measurement *v*, that together can be detected by the set power γ. The lengths of the semi-major and minor axes of the ellipse can be found by eigenvalue decomposition of the positive definite matrix $B=C_{y}^{T}Q_{yy}^{-1}Q_{\hat{e}\hat{e}}Q_{yy}^{-1}C_{y}$, where $Q_{\hat{e}\hat{e}}$ is the variance matrix of the least-squares residuals, and matrix $C_{y}$ specifies, by means of zeros and ones, as an extension to for instance model (6), which image measurement is impacted by the faults ▽u and ▽v.

### 3.3. External Reliability: (Maximum) Horizontal Position Impact Vector Length

A fault with the size as described by the internal reliability in the previous subsection is—by the procedure of statistical testing the measurements—left undetected, with a probability of β=1−γ. The purpose of external reliability is to evaluate how such an undetected fault will propagate into the estimators for the parameters of interest (e.g., position coordinates of the camera in the world frame). For the one-dimensional fault MDB, one can map the (undetected) fault in the measurement $▽y=c_{y}$, into the estimates $▽\hat{x}$ using the well-known Best Linear Unbiased Estimator (BLUE) equation [28]. In this study we focus on the horizontal position bias magnitude in the world frame (a length in [m]), hence on $\sqrt{▽X^{2}+▽Y^{2}}$, when *X* and *Y* are the horizontal position coordinates.

When we consider a fault in *u* and another fault in *v* at the same time, we have two degrees of freedom, and as discussed before, it is not possible to assign a single number as a measure of internal reliability. Instead, the vector ▽ traces the contour of an ellipse, cf. Figure 3. Still we can propagate the two faults together into the estimate for the position. The vector ▽ is decomposed as

(10) $$\begin{array}{c} ▽=∥▽∥\cdot d \end{array}$$

where *d* is a unit direction vector ${\left[\cos\theta ,\sin\theta \right]}^{T}$. We let vector *d* scan the unit circle (numerically, in small steps). For each angle θ, the vector *d* causes vector ▽ to have only one degree of freedom, which can then be solved using the earlier quadratic equation for the non-centrality parameter. For all vectors ▽ in the search, the impact on the horizontal position is computed (length of bias vector), and the maximum over all angles is taken. Note that as the quadratic equation reveals only the magnitude, and not the sign, it is sufficient to have angle θ ranging in [0,π], rather than [0,2π].

## 4. Precision and Reliability Analysis

Theoretical analyses are carried out to evaluate the performance of observation testing and parameter estimation for monocular vision, SF-PPP GNSS, and their integration, in selected scenarios. Also, an extended integration model is considered. For the analyses of these models no actual observations are needed.

The theoretical analysis consists of considering standard deviations of the parameter estimators and the correlation coefficients between these estimators, and the internal and external reliability measures. The MDBs for internal reliability, and the external reliability horizontal position bias vectors are computed for both one and two dimensional fault-cases, both for a nominal case and a reduced case with fewer measurements. Note that reliability relies on measurement redundancy in the system (and hence, in a measurement system without any redundancy, the MDBs will be infinitely large).

### 4.1. System Set-Up

The following camera parameters were set: focal length: 4.9 mm, image size: 3000 × 4000 pixels, and true camera position and attitude: κ = 0.20 rads, $X_{0}$ = 2.800 m, $Y_{0}$ = −0.500 m, $Z_{0}$ = 1.500 m. The vision measurement uncertainty is set to $\sigma_{u}$ = $\sigma_{v}$ = 5 pixels, and the uncertainty in the landmark positions in the HD-map to $\sigma_{X}$ = $\sigma_{Y}$ = $\sigma_{Z}$ = 0.20 m. The focal length and image size correspond to those of a common digital pocket camera. The uncertainty in the vision measurements is rather small (optimal object recognition is assumed, as well as a distortion-free image). The landmark position uncertainty is set to the level of current HD maps for automotive applications (typically a few decimeter).

**Figure 4:**
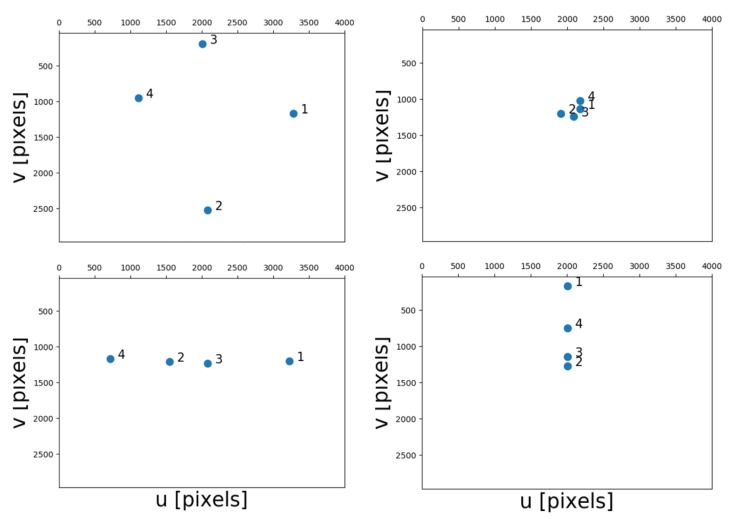
Geometry of 4 selected features in the image plane of four scenarios, with the origin of the image plane at top left. Top left: normal, top right: cluster, bottom left: line up in x, bottom right: line up in y.

The landmark geometry is critical to the quality of the position solution. We consider four scenarios with particularly distributed landmark geometries, see Figure 4. In the ‘normal’ scenario, the landmarks are well distributed in the image plane; in the ‘cluster’ scenario, the landmarks are crowed together; in the ‘line up in x direction’ scenario, the landmarks are aligned around the image horizontal center line; and in the ‘line up in y direction’, the landmarks are ‘standing vertically’ along the central line of the image. The latter three scenarios represent rather extreme cases. All geometries are obtained by changing landmark coordinates in X and Y, leaving the coordinates in Z as constant (in the camera world frame). Scenarios with less landmarks (or features) are obtained by discarding the first landmark, and next the first and second landmark.

### 4.2. Vision Results and Discussion

#### 4.2.1. Precision and Correlation Coefficients

The diagonal values in Table 1 represent the standard deviations of the estimators, whose units are: [degree, m, m, m]. The rest (off diagonal) are the correlation coefficients between the corresponding estimators, cf. (8); they are dimensionless.

From Table 1, the estimates computed by the four scenarios can meet a standard deviation of about 0.5 m for the $X_{0},Y_{0}$ and $Z_{0}$ coordinates of the camera. Standard deviations of κ in the normal and line up in y direction scenarios are below 0.08 degree, which are better than the ones estimated from the cluster and line up in x direction scenarios. The correlation coefficients between the estimates are fairly small.

By inspecting the solutions from the 2-features geometries, in Table 2, the normal and line up in y scenarios are able to solve the camera pose with comparable quality as the 4-features case. In the cluster scenario, the correlation coefficients between κ and $X_{0},\kappa$ and $Y_{0}$ increase, but are still fairly low. The line up in x produces the worst camera pose among the four selected geometries, the standard deviation of κ degrades to 3.74 degree, and the standard deviation of $X_{0}$ and $Y_{0}$ are 0.62 m and 0.59 m respectively. The correlation of κ and the horizontal coordinates increases significantly by discarding 2 features.

#### 4.2.2. Internal Reliability

**Figure 5:**
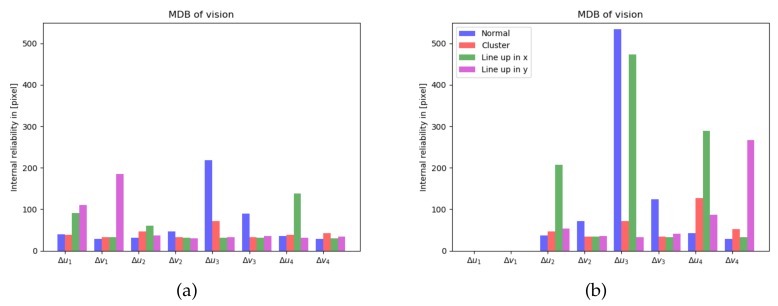
Internal reliability: MDB for a single outlier in vision measurements, computed for 4 features (**a**-left), and for 3 features (**b**-right).

The 1-fault MDBs for the 4 features case are shown in Figure 5a. The 1-fault MDBs for the 3 features case is shown in Figure 5b, and the third and fourth feature get a much larger MDB. Throughout the paper the false alarm probability α and missed detection probability β for the reliability analysis are both set to α = β = 0.005.

#### 4.2.3. External Reliability

**Figure 6:**
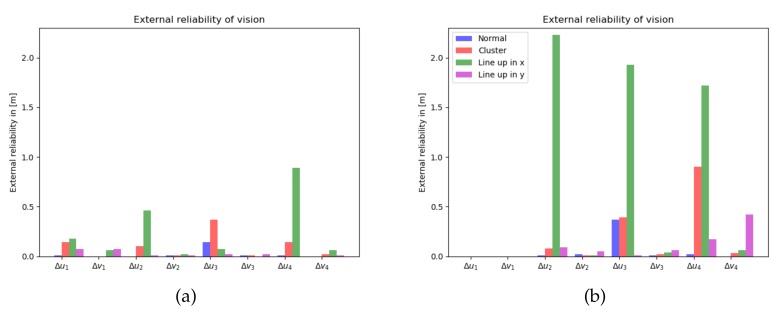
External reliability: magnitude of bias vector in horizontal position, by undetected outlier in vision measurements, computed for 4 features (**a**-left), and 3 features (**b**-right).

The 1-fault external reliability shows by how much the horizontal position estimates get impacted by an undetected fault in a measurement. From Figure 6a, one can see that the bias size is generally at the few decimetres level. By leaving out one feature in the image, Figure 6b, in particular the external reliability bias magnitudes in the line up in x-direction scenario increase to the few meters level.

**Figure 7:**
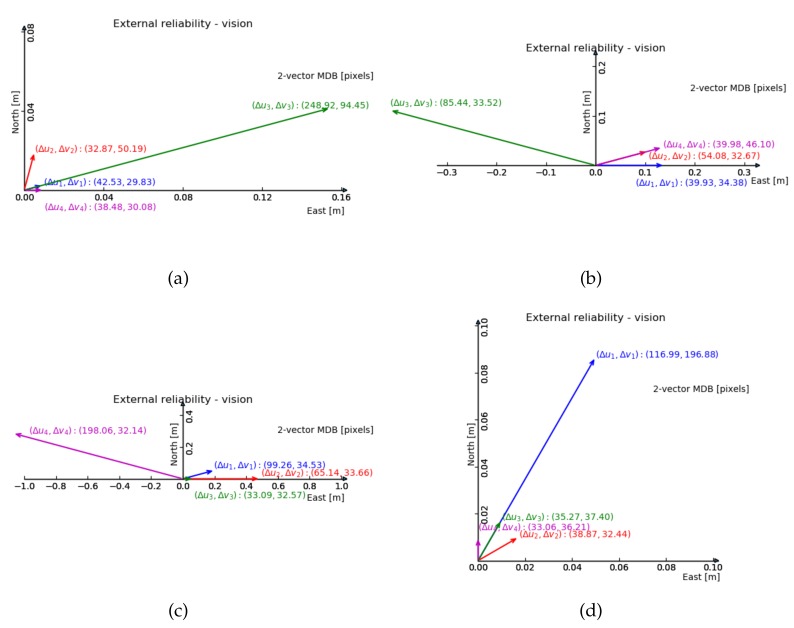
2-fault external reliability computed by 4 features in 4 geometries in [m], the bias vector with largest magnitude is shown (in terms of horizontal position), and the corresponding internal reliability values are given between brackets. (**a**) Normal geometry; (**b**) Cluster; (**c**) Line up in x direction; (**d**) Line up in y direction. Scaling is different for each drawing.

**Figure 8:**
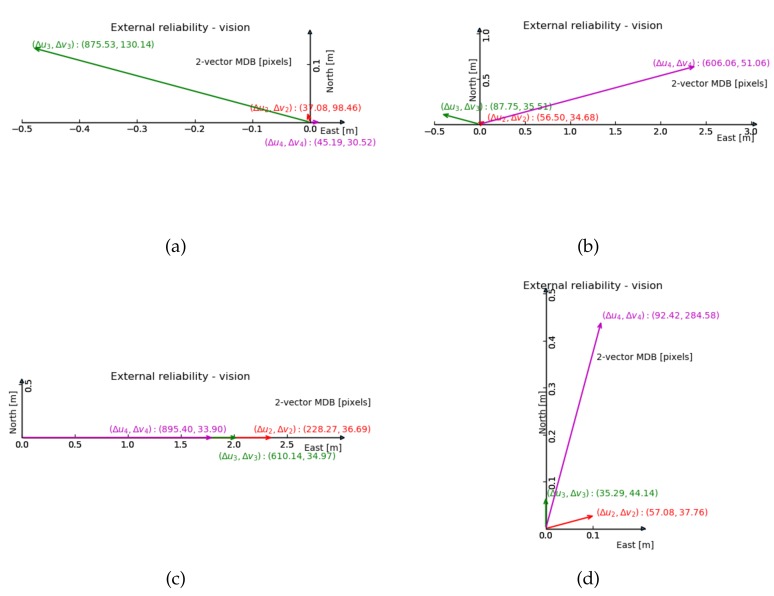
2-fault external reliability computed by 3 features in 4 geometries in [m], the bias vector with largest magnitude is shown (in terms of horizontal position), and the corresponding internal reliability values are given between brackets. (**a**) Normal geometry; (**b**) Cluster; (**c**) Line up in x direction; (**d**) Line up in y direction.

For the 2-fault external reliability, Figure 7 and Figure 8 show the maximum impact of an undetected two-dimensional image fault, on the two-dimensional horizontal position. The line up in y geometry shows the smallest impact.

Comparing Figure 7 and Figure 8, we can see the size of the external reliability vectors in the normal geometry is small, except for the one composed by the third feature (in green). The line up in x geometry suffers most from reducing the amount of features by one, judging by the range of the external reliability.

In summary, the normal geometry has overall best performance, based on evaluation of standard deviation and correlation coefficients of the estimators in the theoretical analysis, then followed by line up in y direction, cluster and finally, line up in x geometry. The cluster and line up in x direction geometries have overall smaller MDBs both for 1-fault and 2-fault; less features give larger MDBs, which is intuitively appealing as there is less validation capability between the observations. The 1-fault MDB has overall smaller values than the 2-fault ones.

### 4.3. GNSS Results and Discussion

#### 4.3.1. Precision and Correlation Coefficients

**Figure 9:**
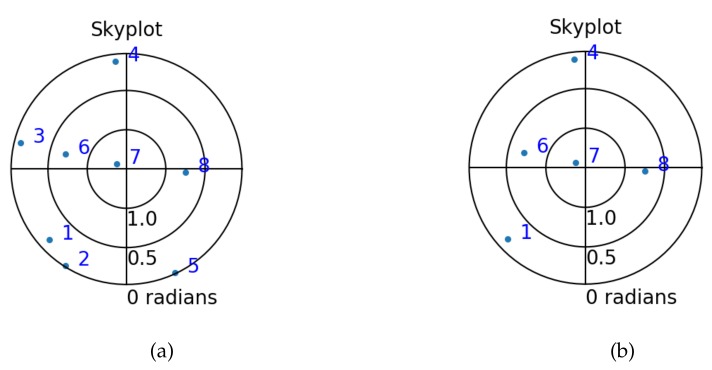
Skyplot with azimuth and elevation of 8 (**a**) and 5 (**b**) satellites for the analysis of SF-PPP GNSS positioning.

The precision of the parameters in Table 3 ranges from 0.78 m to 1.58 m, with relatively high correlation between $Z_{0}$ and $t_{r}$. This is because the satellites are far away from the Earth surface and they are all on one side of the receiver, namely above it. They all contribute to the estimation of the local height component (Z-coordinate) through the sine of their elevation angle (which are all larger than 5 or 10 degrees), and, they all contribute by a coefficient of one to the estimation of the receiver clock error. In addition to these coefficients being similar, higher elevation satellites get a larger weight through the stochastic model, and thereby these two parameters are hard to separate, leading to a large correlation between them. Ultimately, if all satellites were in the local zenith (sine of elevation angle equal to one), it would not be possible to separate them at all. Inspecting the satellite geometry in Figure 9, one can see that the satellites are well distributed, which is responsible for the fair correlation between the other estimates. By reducing the number of available satellites pseudorange observations, the precision of the estimators for the unknown parameters degrades.

Figure 9 shows the skyplot of all available GPS satellites in this simulated experiment. One the left, one can see that the satellites are quite well distributed over the sky.

#### 4.3.2. Internal Reliability

**Figure 10:**
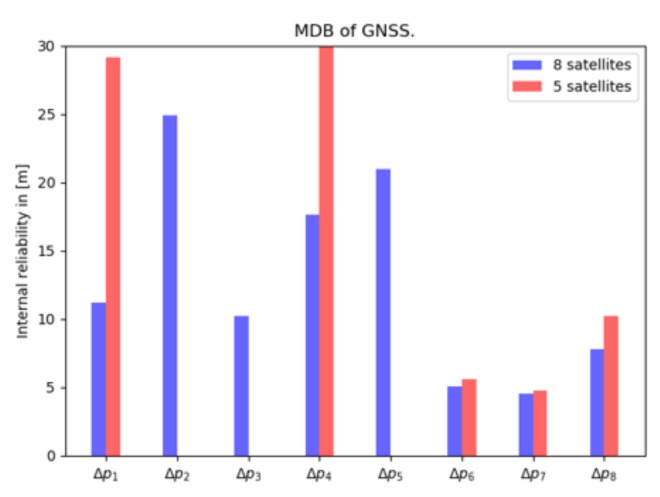
Internal reliability: MDB for a single outlier in GNSS pseudorange measurements, computed for a case with 8 satellites (in blue), and with 5 satellites (in red).

As possible faults in GNSS observations, outliers are considered, and the internal reliability is represented by the 1-fault MDB, see Figure 10. The MDBs for the 8 pseudorange observation case (in blue) vary from 4.5 m to 25 m. The pseudorange observations are selected based on elevation—the three lowest elevation satellites were left out. With only 5 satellites, the values of the MDBs (in red) increase, especially for the first one, the MDB increases by more than 2 times. However, the sixth and seventh pseudorange observation have small values and the increase is also small, which means that the two observations are more reliable and can be well verified with the other observations.

#### 4.3.3. External Reliability

**Figure 11:**
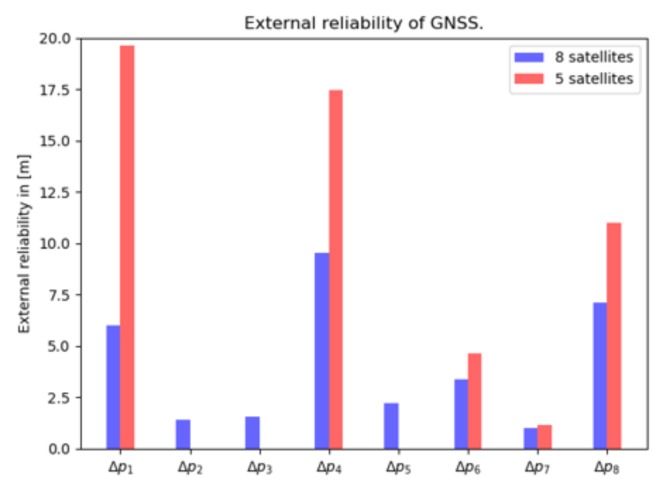
External reliability computed by 8 satellites (in blue) and 5 selected satellites (in red) in [m], presented in the local reference frame.

Figure 11 shows the magnitude of the bias in the horizontal position, when a pseudorange outlier with the magnitude of the MDB remains undetected. This graph closely follows the behavior observed in Figure 10.

In summary, we have seen high correlation between $t_{r}$ and $Z_{0}$, which is inherent to GNSS positioning. By reducing the number of satellite pseudorange observations from 8 to 5, the standard deviations of the unknowns degrade by 20%, but they are still within 2 m. By reducing the number of satellite pseudorange observations, while the number of unknown parameters remain the same, the MDB values increase, as expected, due to the lower redundancy.

### 4.4. Integration Results and Discussion

The integration aims to combine the output from the two sensors, the low-cost GNSS receiver for SF-PPP and the monocular camera. In this section we will use the ‘normal’ vision geometry (cf. Figure 4 top-left).

#### 4.4.1. Precision and Correlation Coefficients

With sufficient satellite pseudorange and image observations, Table 4 shows that the standard deviation of $X_{0},Y_{0},\kappa$ can meet 0.5 m and 0.07 degree respectively. The standard deviation of $Z_{0}$ is 1.5 m, and the correlation coefficients between the three estimators $Z_{0},t_{0},t_{r}$ are close to 1. The system is unable to tell estimators $Z_{0},t_{0}$ and $t_{r}$ from one another. As we explained above, a GNSS receiver cannot tell $Z_{0}$ from $t_{r}$, and the integration system is in addition unable to tell the height offset between the camera and the GNSS antenna $t_{0}$ from $t_{r}$.

When the number of GNSS satellites is reduced from 8 to 2, and the number of features from 4 to 2, the horizontal coordinates still achieve similar precision as in Table 4, though the standard deviations for $Z_{0},t_{0}$ and $t_{r}$ increase to about 6 m (these results are not shown here).

#### 4.4.2. Internal Reliability

**Figure 12:**
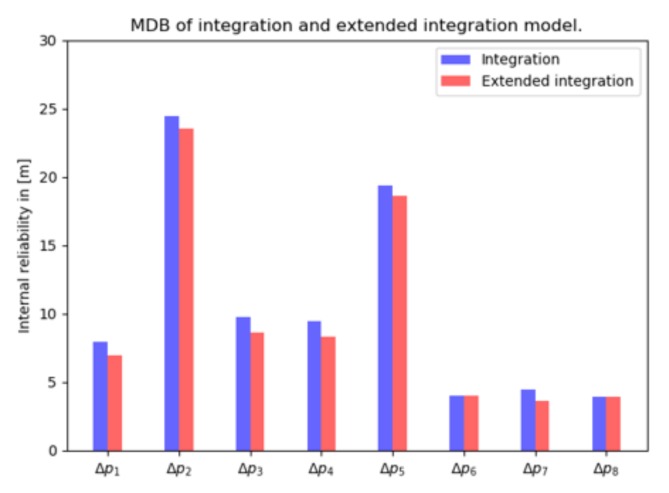
Internal reliability: MDB for a single outlier in GNSS pseudorange measurements, in integration (blue) and extended integration model (red), with 8 satellites and 4 landmarks.

The so-called 1-fault MDB is computed for a scenario with 8 satellite pseudorange observations and 4 detected visual landmarks. Comparing Figure 12 with Figure 10, the MDB values of GNSS pseudoranges decrease thanks to adding the vision observations. The vision MDBs (with normal geometry, in blue) of Figure 5a remain the same in the integration (except for some incidental, very marginal reductions, and are therefore not shown here).

#### 4.4.3. External Reliability

**Figure 13:**
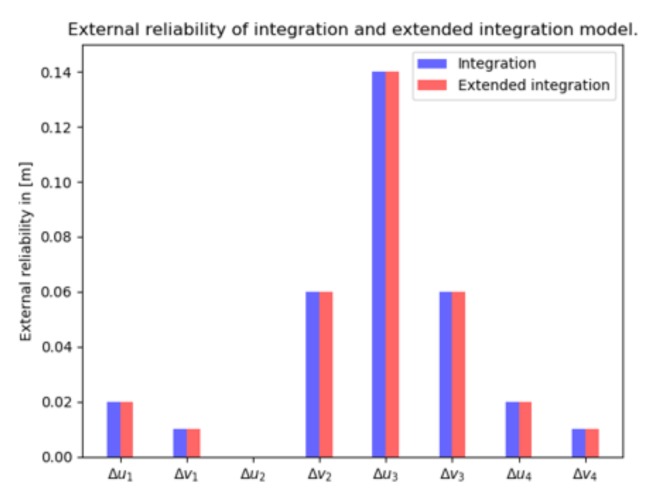
External reliability of vision measurements in the integration (in blue) and extended integration model (in red), with 8 satellites and 4 landmarks.

The vision observations have a much stronger contribution compared to GNSS pseudoranges in the integration model. The vision external reliability vectors in Figure 13 (in blue) are of the same order of magnitude as those in Figure 6a (in blue). The GNSS pseudorange external reliability vectors have magnitudes very close to zero, meaning that undetected faults have very little impact on the (horizontal position) state estimation (and are not shown here).

**Figure 14:**
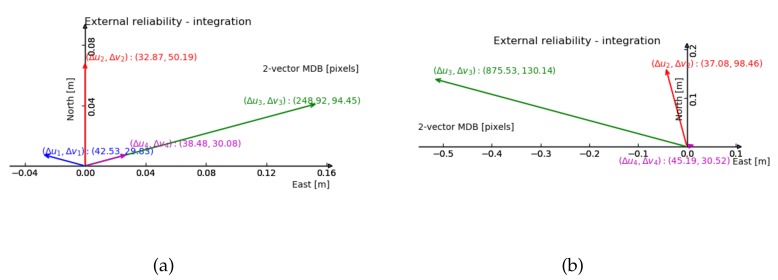
2-vector external reliability of vision measurements in the integration model with 8 satellites and 4 features (**a**), and with 3 satellites and 3 features (**b**). The bias vector with largest magnitude is shown (in terms of horizontal position) and the corresponding internal reliability values are given between brackets.

From the previous 2-fault vision reliability results (Figure 7a and Figure 8a, with normal geometry), one can anticipate that the external reliability of the third feature has the largest value. The results in Figure 14 show that the external reliability is not subject to the input of GNSS observations (it is not much different from Figure 7a). Figure 14b shows the external reliability vectors for the case with 3 satellites and 3 features, which is quite similar to Figure 8a (with 3 features and no satellites).

### 4.5. Extended Integration Results and Discussion

In the so-called extended integration model, we assume to know the (height) offset between the GNSS antenna and the camera.

#### 4.5.1. Precision and Correlation Coefficients

Compared with Table 1, the first 4 columns in Table 5 reach the same performance as the vision-only case. The values in the last column, which describe the correlation coefficients between $t_{r}$ and the other unknowns, and the standard deviation of $t_{r}$ (last one), has significantly declined, cf. Table 4.

Table 6 describes, as an extreme case, when the two sensors both individually fail to produce a position solution. Although the extended integration model is able to produce a position solution, the result is likely far off from the truth; the standard deviations of the estimators are large, especially for $Y_{0}$ and κ. The correlation coefficients between the estimators are close to 1.

#### 4.5.2. Internal Reliability

From Figure 12 (in red) we observe that the GNSS observations have smaller MDBs by leaving out offset $t_{0}$. However, the MDBs computed for the vision observations remain the same (as in Figure 5a), which indicates that the vision observations barely contribute to $t_{0}$ estimation.

#### 4.5.3. External Reliability

In Figure 13 the external reliability computed by 4 vision measurements (in red) are the same as with the integration model (in blue). When the first two features are left out, the remaining vision external reliability vectors grow to the few meters level (not shown here). The vision external reliability vectors (for two faults) are approximately the same for the integration (shown in Figure 14) and extended integration model.

### 4.6. Summary

Compared with GNSS alone positioning, the integration method can reduce the standard deviation of the horizontal position coordinates $X_{0}$ by 50% and $Y_{0}$ by 70%. The extended integration method can further reduce the standard deviation of the vertical position coordinate $Z_{0}$ by 73% and the GNSS receiver clock bias $t_{r}$ by 65%, and in particular reduce the correlation between $Z_{0}$ and $t_{r}$. In terms of reliability, the integration is beneficial to the GNSS observations. The external reliability shows that the system is robust against faults and anomalies. When these remain undetected, at a low probability of missed detection (0.5%), their impact on the horizontal position coordinates is at the 1 decimeter level only. With graphs like Figure 7 and Figure 8, one can analyze in which direction the position will be impacted actually most by undetected faults, and decide whether the designed system satisfies the demands of the application at hand. The integration model is able to produce a position solution when one of the two sensors fails, and theoretically, the extended integration model is able to produce a solution even when both sensors fail. The integration model is able to estimate the height offset $t_{0}$ between the camera and antenna, with high correlation with another two estimators ($Z_{0}$ and $t_{r}$), since vision has little contribution to the $t_{0}$ estimation. In the extended integration, $t_{0}$ is treated as a known constant. Vision measurements can significantly improve the precision of estimators, when satellite pseudorange observations are insufficient. Adding satellite pseudorange observations can only slightly improve the precision of the estimators, because the estimators standard deviation obtained by vision is much smaller than by GNSS, which is also verified by [15]’s experiment.

## 5. Experiment and Result

### 5.1. Experiment Setup

**Figure 15:**
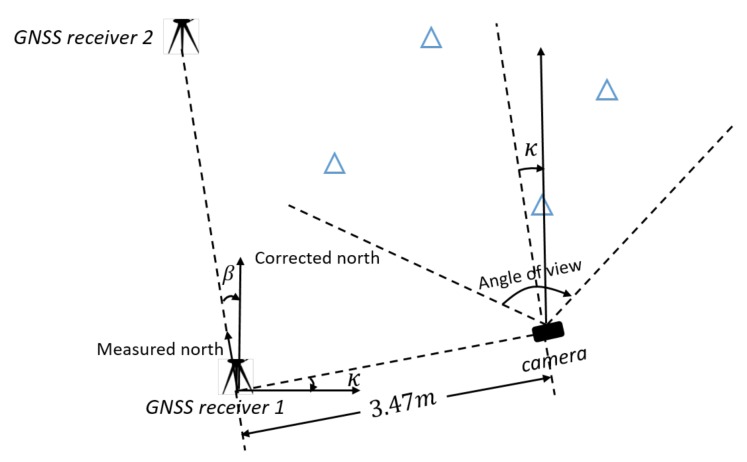
Design of experiment setup. GNSS receiver 1 occupies the origin of the world frame, its North can be related to the ECEF frame by measuring the relative position between the two GNSS receivers. Once the measurements of the two high-end GNSS receiver are completed, GNSS receiver 2 was moved to the camera position, and GNSS receiver 1 was swapped with the total station. The camera is set up locally horizontal and aligned with the world frame surveyed with the total station, about 3.5 m away. Heading κ is obtained by the total station, so that a full ground truth is available for the camera state. The landmarks (blue) are placed within the angle of view and a maximum range of 8 m.

Based on the set-up used in the previous section on theoretical performance analysis, a real experiment is designed and performed, to experience performance of the developed model in practice. The equipment consisted of a low-cost single frequency GNSS receiver, a u-blox EVK-7P, of which GPS and Glonass L1 measurements were used (pseudoranges only in this paper), and a consumer-grade digital camera, a Samsung ES25, delivering the images. The camera has a focal length of 4.9 mm, and an image size of 3000 × 4000 pixels. The GNSS antenna is mounted on top of the camera with a height offset. The geometry of the landmarks is chosen as the normal one according to the earlier analysis (cf. Figure 4), and the positions are measured by a land-surveying total station. The total station is set to be the origin of the local map. In order to determine the local North direction precisely, an extra GNSS receiver was needed to compute the North correction angle, which is the angle between the baseline and actual North, see Figure 15.

The true camera pose was: κ = 3.48 rads, $X_{0}$ = −3.192 m, $Y_{0}$ = −1.360 m, $Z_{0}$ = 1.408 m (used as a ground truth for validation).

**Figure 16:**
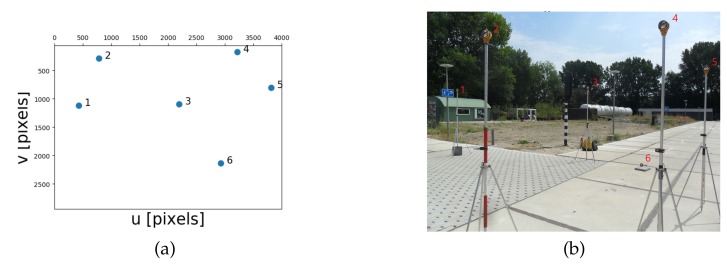
(**a**) Visualization of experiment setup, photograph of the reflectors (landmarks) from the camera perspective (**b**) Image taken by the camera. The image measurements are obtained by manually selecting the geometric centre of the reflectors in the image. The experiment was carried out at the Green Village on the TU Delft campus.

The image measurements are obtained manually, and the landmark positions in the local topocentric system (world frame) are measured by the total station, see Figure 16. The uncertainties for image measurements and landmark positions are set to: $\sigma_{u}$ = $\sigma_{v}$ = 20 pixels, $\sigma_{X}$ = $\sigma_{Y}$ = $\sigma_{Z}$ = 0.20 m (though the latter are actually more precise in this case).

### 5.2. Vision Results and Discussion

The obtained solution is compared with the ground truth values. According to Table 7 the position error is always below 0.2 m, though the error seems to get larger when less reflectors (landmarks) are used.

### 5.3. GNSS Results and Discussion

From Table 8, a single epoch position estimation, when using all satellite pseudorange observations, leads to errors of a few meters. When only pseudorange observations of the 5 highest elevation satellites are used, the $Z_{0}$ degrades significantly, since the selection reduces the variation in vertical direction, and it increases the difficulty for GNSS to estimate $Z_{0}$ together with the receiver clock bias.

### 5.4. Integration Results and Discussion

Table 9 shows that the position error with the integration model is increased significantly when GNSS operates and vision fails (last row). Insufficient vision measurements lead to a poor result for all unknown parameters.

### 5.5. Extended Integration Results and Discussion

Comparing Table 10 with Table 9, the extended integration model improves the estimate of $Z_{0}$ (smaller error) by using all available observations from both sensors. A state estimate can still be computed when both sensors fail at the same time, yet performance is poor.

### 5.6. Summary

Vision observations can significantly improve the GNSS position solution, since the vision solutions have higher precision; they contribute more in the integration model than the GNSS observations. Estimation of the height offset $t_{0}$ relies primarily on the GNSS observations, and camera orientation κ is estimated primarily from the vision observations.

## 6. Conclusions

When pre-surveyed landmark positions are accurate at the 20-cm-level (standard deviation), the integration model is able to estimate the horizontal position coordinates $X_{0},Y_{0}$ within a standard deviation of about 0.5 m, and the vertical position coordinate $Z_{0}$ and the antenna-camera offset $t_{0}$ within about 1.5 m, and heading κ within 0.1 degree, with fair correlation between the unknowns, except between $Z_{0},t_{0}$ and GNSS receiver clock bias $t_{r}$. These unknowns are quite similarly related to the observations, and it is difficult for the integration model to differentiate the observational data over these parameters. We also investigated the performance by leaving out offset $t_{0}$, which is the so-called extended integration model. Then the standard deviations of $Z_{0}$ and $t_{r}$ improve, by eliminating the correlation between $Z_{0}$ and $t_{0},t_{0}$ and $t_{r}$. The integration model is able to produce a position solution when one of the two sensors fails: when GNSS operates and vision fails, the integration can barely improve the GNSS solution. The precision of the estimates is not acceptable, because the vision measurements are insufficient to estimate κ. Including the antenna-camera offset $t_{0}$ also aggregates the correlation between $t_{0},Z_{0}$ and $t_{r}$, and all these rely on the GNSS measurements which can barely produce a position solution. When GNSS fails and vision operates, the integration can improve the GNSS solution significantly, with acceptable precision of the estimates. The extended integration model is theoretically capable of handling more complicated situations: even when the two sensors both fail individually, however, the performance is poor in this extreme case. The reliability of the integrated system is found to be good. The external reliability, with regard to both one and two dimensional faults in the vision measurements, as well as to outliers in the GNSS pseudorange measurements, is only at the 1 decimeter level (at most). This means that the system is well able of detecting faults and anomalies, if there are, and, in the unlikely event that such remains undetected, at a very small probability of missed detection (0.5%), the impact on the position solution is very limited.

The integration model can improve the GNSS position solution in urban areas. In these circumstances, the integrated system can provide a more precise position solution than the one provided by SF-PPP GNSS. This work can be regarded as part of a fully automated GNSS/vision positioning procedure, which breaks the limitations of the two sensors individually, and is more robust to faults and anomalies in challenging environments. Although the project is done in the context of map-based vehicle positioning, the work mainly focuses on the integration performance analysis once pre-processed data (e.g., vision object recognition and data association) from both sensors is obtained. The image processing of the vision part in this work was done manually by human operation. This can be replaced by an automated procedure. As one can see, the errors in the vision observations are much smaller than in the GNSS observations in our experiment, so the integration model is largely constrained by the vision model, and the GNSS model through SF-PPP has little contribution to the final estimates. One way to improve this is to add GNSS carrier phase observations, which can increase the computational load of the integration model, but allow GNSS to contribute much more. Therefore, the integration model is potentially able to produce estimates better than the position solution produced by any one of the sensors. The carrier phase measurements are much more precise than the pseudoranges. They are however ambiguous, though the ambiguity is a constant parameter (in case of uninterrupted signal tracking). The integration model in this research is meant for vehicle single epoch positioning and can work on a snap-shot basis. For real-time positioning in dynamic application, one can use a time series of observations, through for instance an Extended Kalman filter.

## Figures and Tables

**Table 1:** Interpreted variance matrix $Q_{\hat{P}\hat{P}}$ of 4-feature simulated geometries.

	Normal				Cluster				Line up in x				Line up in y			
κ	0.07	0.01	−0.00	−0.00	0.40	0.07	0.15	0.02	0.41	0.12	0.05	0.00	0.08	0.01	0.00	0.00
$X_{0}$		0.45	−0.00	−0.00		0.45	0.00	−0.00		0.45	0.00	0.00		0.45	−0.00	−0.00
$Y_{0}$			0.45	0.00			0.47	0.01			0.45	0.00			0.45	0.00
$Z_{0}$				0.45				0.45				0.45				0.45

**Table 2:** Interpreted variance matrix $Q_{\hat{P}\hat{P}}$ of 2-feature simulated geometries.

	Normal				Cluster				Line up in x				Line up in y			
κ	0.18	0.01	0.05	0.02	0.44	0.08	0.17	0.02	3.74	0.69	0.65	0.04	0.29	0.05	0.01	0.00
$X_{0}$		0.45	−0.00	−0.00		0.45	−0.01	−0.00		0.62	0.45	0.03		0.45	−0.01	−0.00
$Y_{0}$			0.45	0.00			0.50	0.03			0.59	0.03			0.45	0.01
$Z_{0}$				0.45				0.45				0.45				0.45

**Table 3:** Interpreted variance matrix $Q_{\hat{P}\hat{P}}$ from all available 8, and 5 satellite pseudorange observations. The unit of $X_{0}$, $Y_{0}$, $Z_{0}$, $t_{r}$ is all [m].

	8 Satellites				5 Satellites			
$X_{0}$	0.78	0.06	0.36	0.41	0.83	0.05	0.27	0.31
$Y_{0}$		1.41	0.07	0.04		1.25	0.06	0.09
$Z_{0}$			1.58	0.97			1.91	0.98
$t_{r}$				1.36				1.68

**Table 4:** Interpreted variance matrix $Q_{\hat{P}\hat{P}}$ computed from pseudorange observations of 8 satellites and image observations of 4 features, with integration model, units are [m, m, m, degree, m].

	Normal					
$X_{0}$	0.39	0.01	0.19	0.18	0.01	0.22
$Y_{0}$		0.42	0.02	0.02	0.00	0.01
$Z_{0}$			1.49	0.96	0.00	0.93
$t_{0}$				1.56	0.00	0.93
κ					0.07	0.00
$t_{r}$						1.27

**Table 5:** Interpreted variance matrix $Q_{\hat{P}\hat{P}}$ computed from pseudorange observations of 8 satellites and image observations of 4 featutres with extended integration model, units are [m, m, m, degree, m].

	Normal				
$X_{0}$	0.38	0.01	0.06	0.01	0.13
$Y_{0}$		0.42	0.01	0.00	−0.03
$Z_{0}$			0.43	−0.00	0.74
κ				0.08	0.00
$t_{r}$					0.48

**Table 6:** Interpreted variance matrix $Q_{\hat{P}\hat{P}}$ computed from pseudorange observations of 3 satellites and image observations of 1 feature with extended integration model, units are [m, m, m, degree, m].

	Normal				
$X_{0}$	2.98	0.96	0.95	0.99	0.93
$Y_{0}$		18.98	0.99	0.97	0.97
$Z_{0}$			3.19	0.96	0.99
κ				18.41	0.94
$t_{r}$					2.14

**Table 7:** Error of camera state estimation by vision model, units are [degree, m, m, m].

Scenarios	κ	$X_{0}$	$Y_{0}$	$Z_{0}$
6 reflectors	−0.10	−0.00	0.03	−0.02
5 reflectors	0.07	−0.01	0.02	−0.02
3 reflectors	0.03	0.01	0.14	−0.06
2 reflectors	−0.86	0.09	0.16	−0.07

**Table 8:** Actual error of position estimation by GNSS model, units are [m, m, m].

Scenarios	$X_{0}$	$Y_{0}$	$Z_{0}$
13 satellites	3.07	1.66	2.91
5 satellites	2.44	4.62	−17.70

**Table 9:** Actual error of camera state estimation by integration model, units are [degree, m, m, m].

Scenarios	κ	$X_{0}$	$Y_{0}$	$Z_{0}$
13 satellites and 6 features	0.24	0.96	0.50	1.62
3 satellites and 2 features	−0.83	−0.05	0.39	−8.02
4 satellites and 1 features	13.32	−3.43	6.34	−54.50

**Table 10:** Actual error of camera state estimation by extended integration model, units are [degree, m, m, m].

Scenarios	κ	$X_{0}$	$Y_{0}$	$Z_{0}$
13 satellites and 6 features	0.31	0.93	0.48	0.54
3 satellites and 1 features	−1675.62	103.98	72.61	−23.83
